# Non-Functional Trace Amine-Associated Receptor 1 Variants in Patients With Mental Disorders

**DOI:** 10.3389/fphar.2019.01027

**Published:** 2019-09-13

**Authors:** Grazia Rutigliano, Julia Bräunig, Claudia Del Grande, Vittoria Carnicelli, Isabella Masci, Sergio Merlino, Gunnar Kleinau, Luca Tessieri, Simone Pardossi, Sarah Paisdzior, Liliana Dell’Osso, Heike Biebermann, Riccardo Zucchi

**Affiliations:** ^1^Scuola Superiore Sant'Anna, Pisa, Italy; ^2^National Research Council (CNR), Institute of Clinical Physiology (IFC), Pisa, Italy; ^3^Charité – Universitätsmedizin Berlin, corporate member of Freie Universität Berlin, Humboldt-Universität zu Berlin, and Berlin Institute of Health, Institute für Experimentelle Pädiatrische Endokrinology, Berlin, Germany; ^4^Department of Clinical and Experimental Medicine, Section of Psychiatry, University of Pisa, Pisa, Italy; ^5^Department of Pathology, University of Pisa, Pisa, Italy; ^6^Charité – Universitätsmedizin Berlin, corporate member of Freie Universität Berlin, Humboldt-Universität zu Berlin, and Berlin Institute of Health, Institute of Medical Physics and Biophysics, Group Protein X-ray Crystallography and Signal Transduction, Berlin, Germany

**Keywords:** trace amine associated receptor 1, major mental disorders, single-nucleotide variants, 3-iodothyronamine, Gs/adenylyl cyclase activation, tridimensional homology model

## Abstract

**Background:** The G protein–coupled receptor (GPCR) trace amine-associated receptor 1 (TAAR1) is expressed across brain areas involved in emotions, reward and cognition, and modulates monoaminergic and glutamatergic neurotransmissions. TAAR1 is stimulated with nanomolar affinity by 3-iodothyronamine (T1AM), an endogenous messenger considered a novel branch of thyroid hormone signaling. The human gene for TAAR1 maps to locus 6q23, within a region associated with major mental disorders.

**Materials and Methods:** We screened a cohort of patients with major mental disorders (n = 104) and a group of healthy controls (n = 130) for TAAR1 variants. HEK293 cells were transiently transfected with: i) wild-type TAAR1 and ii) mutated TAAR1, either in homozygous or heterozygous state. Cell surface expression and Gs/adenylyl cyclase activation upon administration of β-phenylethylamine (PEA), T1AM, and RO5166017, were assessed.

**Results:** We detected 13 missense variants in TAAR1 coding region, with a significant enrichment in patients as compared to healthy controls (11 *vs.* 1, 1 variant in both groups, p < 0.01). *In silico* analysis identified four dysfunctional variants, all in patients. Three of these—R23C, Y131C, and C263R—were functionally characterized. In cells co-transfected with wild-type and mutated TAAR1, we observed a significant reduction of cell surface expression. In heterozygosity, the three TAAR1 variants substantially dampened Gs signaling in response to PEA, and, more robustly, to T1AM. Co-stimulation with PEA and RO5166017 did not yield any improvement in Gs signaling. R23C, Y131C, and C263R are rare in the general population and map in functionally important highly conserved positions across TAAR1 orthologous and paralogous genes.

**Conclusions:** Our findings suggest that disruptions of TAAR1 activity may be relevant to the pathophysiology of mental disorders, thereby providing a promising target for novel psychopharmacological interventions.

## Introduction

The G protein–coupled (GPCR) trace amine-associated receptor 1 (TAAR1) belongs to the trace amine receptor family and is widely distributed across vertebrate phyla (Grandy, 2007). So far, its role in physiology and especially in neuromodulation remains elusive. TAARs own their name to their first-described ligands, namely, trace amines, such as β-phenylethylamine (PEA) and p-tyramine (TYR). These are prominent neuromodulators in insects (Grohmann et al., 2003) and modulate the effects of other neurotransmitters in the synaptic cleft in the mammalian central nervous system (CNS) (Berry, 2004). It was later discovered that TAAR1 is also activated by thyronamines, in particular by 3-iodothyronamine (T1AM) (Scanlan et al., 2004), an endogenous messenger thought to derive from thyroid hormone. T1AM was detected in rodent and human tissues (Hoefig et al., 2016) and was found to have several effects in the CNS. Pharmacological application of T1AM in rodents modifies food intake and sleep pattern composition and yields pro-learning and anti-amnestic effects (Manni et al., 2013). TAAR1 signals *via* activation of the Gs/adenylyl cyclase system (Borowsky et al., 2001).

TAAR1 expression has been reported in an array of brain areas, traditionally involved in the physiopathology of cognition, reward, and emotions, such as the ventral tegmental area, the dorsal raphe nucleus, the amygdala, the hippocampus and parahippocampal regions, the prefrontal cortex, and other cortical areas (reviewed in (Rutigliano et al., 2017)). In mono-aminergic nuclei, TAAR1 engages in a cross-talk with the dopaminergic and serotonergic systems, at the level of receptors and transporters. For instance, the formation of heterodimers between TAAR1 and the dopamine receptor 2 (D2R) has been observed (Espinoza et al., 2011; Harmeier et al., 2015) and allegedly leads to recruitment of β-arrestin 2 and silencing of the GSK3β (Harmeier et al., 2015), a pathway known to be involved in psychosis and mood disorders (Willi and Schwab, 2013) and targeted by lithium treatment (Muneer, 2017). Similarly, TAAR1 and the dopamine and serotonin transporters (DAT and SERT) are co-expressed and appear to be reciprocally regulated (Xie and Miller, 2009). A further insight into the role of TAAR1 in the CNS comes from TAAR1 knockout (KO) mice. These, notwithstanding a grossly normal phenotype, show perseveration, impulsivity (Espinoza et al., 2015), impaired sensorimotor gating (Wolinsky et al., 2007), and worse performances in cognitive tests (Achat-Mendes et al., 2012). Also, TAAR1-KO mice display enhanced sensitivity to the addictive effects of amphetamines and ethanol (Achat-Mendes et al., 2012; Lynch et al., 2013). Their behavioral phenotype corresponds to neurochemical and electrophysiological alterations suggesting increased dopaminergic tone (Lindemann et al., 2008; Leo et al., 2014) and dysregulated cortical glutamate transmission (Espinoza et al., 2015). Several synthetic TAAR1-selective agonists have been developed (Rutigliano et al., 2017) and found to produce cognitive effects, to block stress-induced hyperthermia, and to dampen dopamine-driven hyperlocomotion, consistently with anxiolytic- and antipsychotic-like properties (Schwartz et al., 2018).

Taken together, these preclinical findings point to TAAR1 as a promising target of novel pharmacological interventions for mental disorders. However, to date, there is no evidence of an association between disruption of TAAR1 functions and mental disorders. A recent study identified a rare missense variant in TAAR1 (C182F) in three affected members of a small schizophrenia family, and six more variants in sporadic schizophrenia cases (John et al., 2017). However, the functional impact of these rare variants was only assessed with *in silico* prediction tools. Here, we screened a cohort of patients with major mental disorders for TAAR1 variants, and we analyzed the potential molecular function of the mutated amino acid side chains by using a three-dimensional (3-D) TAAR1 homology model (Muhlhaus et al., 2017). Furthermore, we performed functional *in vitro* characterization for a subset of the identified variants, using novel methods that allow the real-time and live-cell assessment of the spatiotemporal dynamics of cell surface expression and Gs signaling. Our findings indicate that TAAR1 variants with deleterious functional effects may contribute to the etiopathology of mental disorders, supporting the potential relevance of this receptor in the physiology of the CNS.

## Materials and Methods

### Sample Recruitment and Psychopathology Assessment

We recruited a consecutive sample of 104 patients aged 18 to 65 years of Caucasian ethnicity, receiving treatment for major mental disorders, including schizophrenia spectrum disorders, bipolar and related disorders, depressive disorders, anxiety disorders, obsessive-compulsive and related disorders, feeding and eating disorders, trauma and stressor-related disorders, externalizing disorders, and substance/alcohol use disorders, at the Psychiatry Unity of the University of Pisa. As healthy controls, we recruited 130 voluntary blood donors at the Division of Transfusion and Transplant Biology, Azienda Ospedaliero—Universitaria Pisana. Subjects were excluded if there was a history of severe medical or neurological disorders, estimated IQ < 60, acute intoxication, and pregnancy. In addition, current or past history of any mental disorder applied as exclusion criterion for healthy controls. Consensus diagnosis was either established or excluded according to the Structured Clinical Interview for DSM-5, Research Version (SCID-5-RV) (First et al., 2015). Socio-demographic characteristics were collected, and psychopathology was quantitatively investigated in patients with: (1) the Positive and Negative Syndrome Scale (PANSS) (Kay et al., 1987), (2) the Hamilton Rating Scale for Depression (HDRS) (Hamilton, 1960), (3) and the Young Mania Rating Scale (YMRS) (Young et al., 1978).

Written informed consent was obtained from the participants to use biological samples and data about clinical measures and treatment. Genetic data has been used in agreement with the Authorization n. 8/2016—general authorization for the processing of genetic data—of 15/12/2016. The study was approved by the institutional ethical committee (Protocol n° 55951, 12/09/2017).

### Screening for TAAR1 Variants

Patients and healthy controls donated 5 ml saliva and 3 ml blood, respectively. Genomic DNA was extracted from biological samples using Quick-DNA Universal Kit (Zymo Research, CA, U.S.A.), according to the manufacturer’s instructions. PCR amplification of three partially overlapping amplicons spanning TAAR1 coding region and the 5’- and 3’-untranslated region (UTR) was performed by using the following three primer pairs (Thermo Fisher Scientific): 5’ AACTCACCATACATACTTTGACTCAAG 3’ (forward primer) and 5’ CTTATCGCTAAAGAACAGGCAAGA 3’ (reverse primer) for the first amplicon of 511 pb, 5’ CAGCTTTCCTTTCTTTGCTTTGTGA 3’ (forward primer) and 5’ TATGGTGAGATCTGCTGAGCACTGT 3’ (reverse primer) for the second amplicon of 509 pb, and 5’ AGACAAATGGAAAATGGAGGCTGAG 3’ (forward primer) and 5’ GGACTCAAATTGCCAATGATTTACTCT 3’ (reverse primer) for the third amplicon of 515 pb. The primer pairs contained the sense and antisense sequences of the universal primers M13. The PCR products were run on a 2% agarose gel, quantified using the Qubit Fluorometer combined with the Quanti-iT dsDNA BR Assay Kit (Invitrogen), and purified using the CleanSweep^™^ PCR Purification Reagent (Applied Biosystems). The purified PCR products were sequenced according to the Sanger method, using the BigDye Terminator Sequencing Kit v1.1 (Applied Biosystems) with the universal primers M13 (Thermo Fisher Scientific). Upon purification with the Mag-Bind SeqDTR Kit (Omega Bio-Tek), the sequences were analyzed by capillary electrophoresis on ABI310 Genetic Analyzer (Applied Biosystems), aligned to the reference gene sequence, and screened for single-nucleotide polymorphisms/variants (SNPs/SNVs), using the online programs GeneScreen (http://dna.leeds.ac.uk/genescreen/download.php) and ABI Chroma Align (http://www.bmr-genomics.it/seq_index.html).

The minor allele frequency (MAF) was calculated for the SNPs/SNVs detected in our sample and compared to the MAF in the general population, as documented in public database (NCBI, dbSNP). We manually aligned human TAAR1 sequence to several TAAR1 orthologous and other human TAAR family members, for assessing the evolutionary conservation of amino acid residues. The alignment was visualized with the software BioEdit and scored using the BLOSUM62 matrix. Three *in silico* tools available in dbNSFP2.9 were used to predict the functional effect of the detected SNPs/SNVs: SNP&GO, http://snps-and-go.biocomp.unibo.it/snps-and-go/index.html; SNAP, https://rostlab.org/services/snap; and PhD-SNP, http://snps.biofold.org/phd-snp/phd-snp.html.

### Structural Human TAAR1 Homology Model

The TAAR1 homology model was designed as previously described (Kleinau et al., 2011). Docking of 3-T1AM into the TAAR1 model was performed as described recently (Braunig et al., 2018).

### Cloning of TAAR1 Wild-Type and Variants

To functionally characterize TAAR1 variants, wild-type TAAR1 (TAAR1-WT) was cloned in the eukaryotic expression vector pcDps (provided by Torsten Schöneberg, University of Leipzig, Germany) (Kleinau et al., 2011). To enhance surface membrane expression, we added TAAR1 with a N-terminus tag encompassing the first nine amino acids of the β2-adrenoreceptor (ADRB2). The β-TAAR1 construct, hereinafter TAAR1, has been previously described to show comparable signaling properties (Barak et al., 2008; Braunig et al., 2018).

For determination of cell surface expression, we introduced TAAR1-WT into the vector pBiT3.1-N (Promega, Mannheim, Germany) *via EcoR*I and *BamH*I restriction sites. pBiT3.1-N contains the HiBiT, a N-terminally positioned 11 amino acid peptide tag (Promega, Mannheim, Germany), which was detected as detailed below. The glucagon-like peptide receptor 1 (GLP1R) was cloned into the pBiT3.1-secN, replacing the signal peptide with the interleukin (IL-6) signal peptide followed by the HiBiT tag, and served as assay control.

Three TAAR1 variants (R23C, Y131C, and C263R) were introduced into TAAR1 by site-directed mutagenesis, using the PfuTurbo DNA Polymerase (Stratagene, La Jolla, CA, United States).

All constructs were sequenced for verification with BigDye-terminator sequencing (Applied Biosystems) and an automatic sequencer (ABI 3710xl; Applied Biosystems, Foster City, CA).

### Cell Culture and Transfection

HEK293 cells were maintained in Minimum Essential Media (MEM, Biochrom GmbH, Berlin, Germany) supplemented with 5% fetal calf serum (FCS) and non-essential amino acids (Biochrom AG, Berlin, Germany), in humidified air at 37°C and 5% CO2. Cells were seeded in poly-L-lysine coated (Biochrom GmbH, Berlin, Germany) 96-well assay plates, at a density of 1.5 × 10^5^ cells/ml. After 24 h, cells were transiently transfected using METAFECTENE (0.45 μl/well), in supplement-free Advanced MEM (Life Technologies, Carlsbad, CA, USA). For the HiBiT assay, cells were transfected with the pcDNA3 empty vector (mock), TAAR1-WT, or co-transfected with TAAR1-WT and variants to resemble the heterozygous state, as well as GLPR as a positive control. For the GloSensor^™^ cAMP assay, cells were co-transfected with TAAR1-WT and variants and the GloSensor plasmid F22 (Promega, Mannheim, Germany). As a negative control, TAAR1 was exchanged with empty vector (mock). In order to mimic the heterozygous state of the variants, TAAR1-WT and mutants were transfected in equimolar plasmid amounts.

### Quantification of Cell Surface and Total Expression

The Nano-Glo^®^ HiBiT extracellular detection system (Promega, Mannheim, Germany) provides an antibody-free, live-cell, highly sensitive method to assess surface expression and internalization. The extracellular HiBiT tag is a subunit of a genetically improved luciferase. A highly sensitive luciferase can assemble by adding the cell-impermeable LgBiT compound. The overall luciferase activity allows conclusions about the surface expression of the tagged protein. We investigated the TAAR1-WT and variants for their cell surface expression. Forty eight hours after transfection, the medium was exchanged to OptiMEM without phenol red (Life Technologies, Carlsbad, CA, USA), and cells were incubated for 30 min at 37°C. We proceeded with the manufacturer alternative protocol for rapid measurements. Following a 4-min incubation with the substrate/LgBiT compound mixture, bioluminescence was quantified using a Berthold Microplate Reader (Berthold Technologies GmbH & Co. KG, Bad Wildbad, Germany). The signal from each well was normalized over the empty pcDNA3 vector. As positive control, we used the HiBiT-tagged GLPR.

A similar approach was used to assess total protein expression using the Nano-Glo^®^ HiBiT Lytic Detection System (Promega, Mannheim, Germany) in combination with the extracellular system. The lytic reagent is able to lyse the plasma membrane so that the total amount of HiBiT-tagged receptors can be measured. Forty eight hours after transfection, the medium was exchanged to Opti-MEM without phenol. Then, we proceeded with the manufacturer protocol for comparing Extracellular and Lytic HiBiT Signals. Due to the different buffer conditions, the signals cannot be compared directly, even when measured simultaneously. Therefore, we compared the signals of a HiBiT control protein (Promega) that is 100% extracellular. The fraction of receptor on the cell surface compared to the total amount of protein expressed was acquired using the following formula:

$$\frac{corr.\;cell\;surf.\;expression_{sample}}{corr.\;total\;expression_{sample}}\times \frac{corr.\;total\;expression_{Ctrl\;prot}}{corr.\;cell\;surf.\;expression_{Ctrl\;prot}}$$

### Measurement of Gs/Adenylyl Cyclase Activation

Live-cell cAMP formation was measured using the GloSensor^™ ^cAMP system (Promega, Mannheim, Germany). Two days after transfection, cells were pre-equilibrated with GloSensor cAMP Reagent (2% in medium containing 88% CO2-independent medium and 10% FKS) in dark at room temperature for 2 h. Bioluminescence, in terms of relative light units (RLU), was quantified using a Berthold Microplate Reader (Berthold Technologies GmbH & Co. KG, Bad Wildbad, Germany). Before ligand challenge, plates were measured six times at 2-min intervals/well to obtain the basal state of TAAR1-WT and variants. For normalization purposes, mock-transfected cells were used. Cells were stimulated with PEA 10 μM (Sigma– Aldrich, St. Louis, MO, USA), T1AM 10 μM (Santa Cruz Biotechnology, Dallas, TX, USA), RO5166017 10 μM (kindly provided by Dr Gainetdinov), or phosphate-buffered saline (PBS) as negative control, and plates were read 19 times at 2-min intervals. The concentration of TAAR1 agonists was chosen based on former cAMP accumulation assays performed to functionally characterize CHO-K1 cells transfected with the human TAAR1 (Coster et al., 2015). The area under the time-response curve (AUC) of cAMP concentration-dependent increase in RLU was assessed as total cAMP formation. As a positive control, we added isoproterenol 10 μM to HEK293 cells transfected with the empty vector, which are known to endogenously express β-adrenergic receptors (Schonbrunn and Steffen, 2012).

To validate the results of the GloSensor^™^ cAMP assay, we replicated the determination of Gs signaling using the AlphaScreen™ technology (PerkinElmer Life Science, Boston, MA, USA) (Muhlhaus et al., 2017). To this purpose, cAMP accumulation was measured upon 45-min incubation with increasing concentrations of T1AM (100 to 10 μM) in presence of isobutylmethylxanthine (IBMX).

### Statistical Analysis

Socio-demographic and clinical characteristics were described using: absolute and relative frequencies for categorical variables, mean and standard deviation (SD), or median and percentiles for continuous variables of normal or non-normal distribution, respectively. Group comparison was performed through Pearson’s chi-squared test or Fisher’s exact test, when appropriate, for categorical variables; and Student’s t-test or Mann–Whitney test, for continuous variables, using SPSS (Statistical Package for Social Sciences), Statistics Version 23.

Data resulting from the *in vitro* assays was visualized and analyzed using one-way ANOVA, followed by Dunnett’s *post hoc* tests in GraphPad Prism 6 (GraphPad Software Inc., La Jolla, CA, USA). Statistical significance was set at *p ≤ 0.05, **p ≤ 0.01, ***p ≤ 0.001, and ****p ≤ 0.0001. Network analysis was run with RStudio, built up of the: PANSS subscales measuring positive and negative psychotic symptoms, and general psychopathology; the three YMRS factors reported by Grover (2018) (factor 1: items 1 to 3; factor 2: items 5 to 9; factor 3: items 10 and 11); and the four HDRS components meta-analytically found by Shafer (2005) (depression: items 1, 2, 3, 7, 8; anxiety: items 9, 10, 11, 15, 17; insomnia: items 4, 5, 6; somatic symptoms: items 12, 13, 14, 16). We followed the methods described in the tutorial paper from Epskamp et al., 2017 (Epskamp et al., 2018). In brief, we estimated undirected edges and centrality indices of nodes (node strength, closeness, and betweenness) using a pairwise Markow random field network model. As data was not normally distributed, we applied the nonparanormal transformation. To retain only the most solid edges, we used a “least absolute shrinkage and selection operator” regularization, setting the Extended Bayesian Information Criterion to 0.5. Centrality indices of nodes were re-measured after case-dropping subset bootstrapping, for assessing their stability, and the correlation stability (CS) coefficient was calculated. The accuracy and 95% confidence interval (CI) of edges were measured *via* nonparametric bootstrapping (Epskamp and Fried, 2016).

## Results

### Socio-Demographic and Clinical Characteristics

Our sample included 104 patients and 130 healthy controls. Relative to healthy controls, patients: were older (40.37 ± 12.49 years *vs.* 36.67 ± 11.54 years; t = −2.35, p < 0.05); were more frequently female (n = 54, 51.9% *vs.* n = 50, 38.5%; χ2 = 4.24, p < 0.05); had a lower education level (graduation and post-graduation n = 24, 23.1% *vs.* n = 65, 50%, χ2 = 17.77, p < 0.001); were more frequently unemployed (n = 21, 20.2% *vs.* n = 4, 3.1%, χ2 = 26.28, p < 0.001); had higher rates of family history of mental disorders (n = 84, 80.8% *vs.* n = 44, 34.1%; χ2 = 50.64, p < 0.001), and more frequently had individual history of perinatal/psychomotor development problems (n = 9, 8.7% *vs.* n = 2, 1.5%; χ2 = 6.53, p < 0.05).

**Figure 1:**
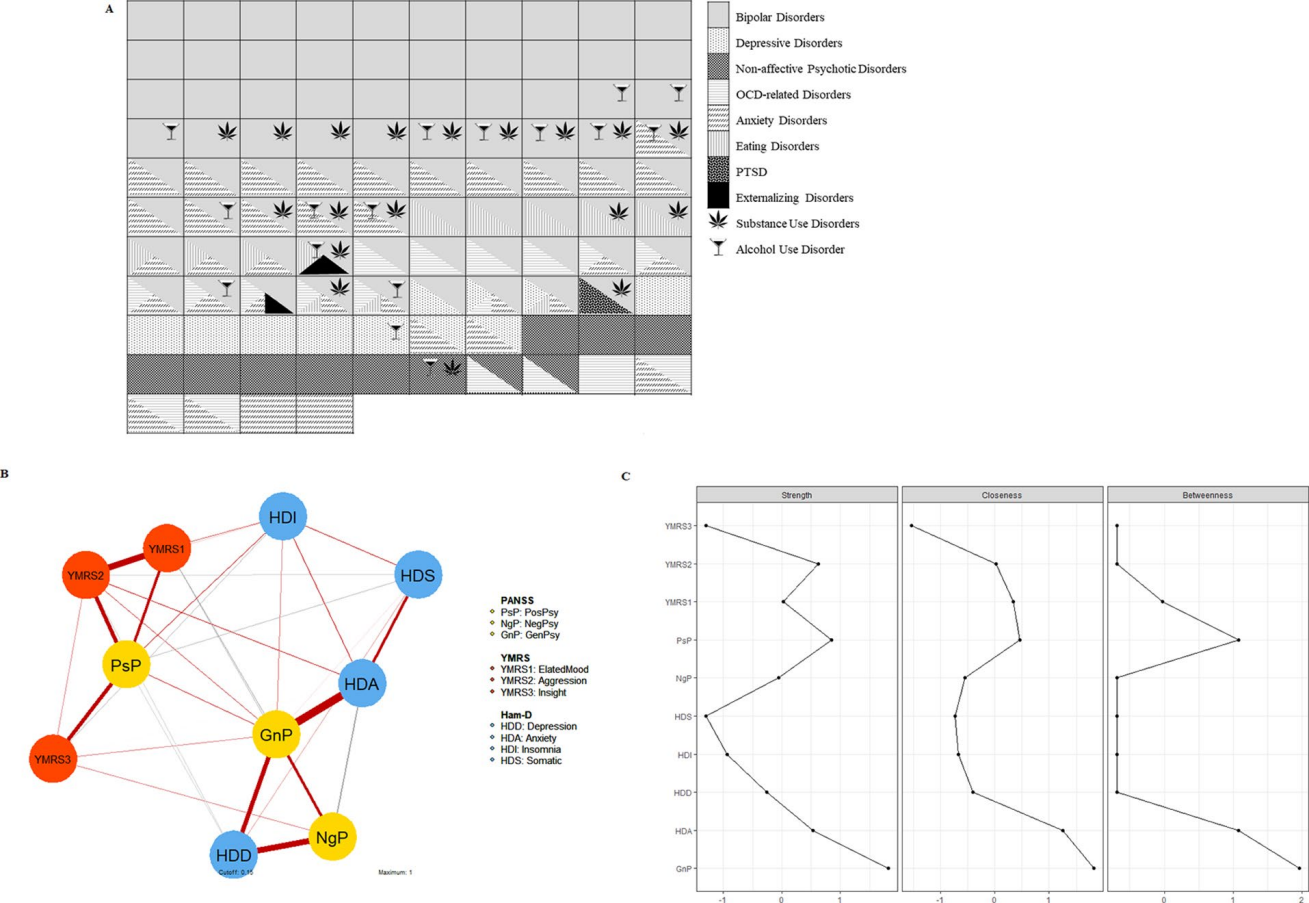
**(A)** Graphical representation of main and comorbid diagnoses in patients. Each of the boxes represents one patient, and it is colored/patterned according to his/her main diagnosis, as established with the SCID-5-RV. Comorbid diagnoses are depicted with overlapping patterned triangles/symbols, as per legend. In the majority of cases (58%), at least two diagnoses co-existed in the same patient. **(B)** Description of the structure of psychopathology in patients *via* network analysis. Each node of the network refers to the scores of a symptom cluster quantified with internationally validated psychometric scales (PANSS, YMRS, HDRS). Red edges indicate positive correlation; gray edges indicate negative correlation. The thickness of edges represents the size of the correlation coefficient. **(C)** Centrality indexes of the psychopathology network. Node strength refers to the sum of weight of the connections for each node. Closeness is the inverse of the sum of the distances of the focal node from all the other nodes in the network. Betweenness is the number of shortest paths between any two nodes that pass through the node of interest. The node with the highest centrality was the PANSS general psychopathology. PsP, PANSS positive; NgP, PANSS negative; GnP, PANSS general; YMRS1, items 1 to 3; YMRS2, items 5 to 9; YMRS3, items 10 and 11; HDD, HDRS depression, items 1, 2, 3, 7, 8; HAD, HDRS anxiety, items 9, 10, 11, 15, 17; HDI, HDRS insomnia, items 4, 5, 6; HDS, HDRS somatic symptoms, items 12, 13, 14, 16.

The majority of patients suffered from mood disorders, either bipolar (BD, n = 79, 76%, of which: BD-I, n = 30, 28.8%; BD-II, n = 34, 32.7%; BD-other, n = 15, 14.4%) or depressive disorders (n = 11, 10.6%). Main and comorbid diagnoses are depicted in **Figure 1A**. The scores (median values and 25^th^–75^th^ percentiles) relative to current psychopathology were: (i) PANSS positive, 9 (7–11); negative, 7.5 (7–13); general psychopathology, 28 (23.25–38); (ii) YMRS, 6 (2–11.75); and (iii) HDRS, 7.5 (4–12.75) (**Table S1**). The network structure of psychopathology is depicted in **Figure 1B**, together with the plot of centrality indices of the nodes (**Figure 1C**). The node with the highest centrality was the PANSS general psychopathology. The largest (>0.5) correlations were between PANSS positive scores and YMRS scores, and between PANSS negative and general psychopathology scores and the HDRS dimensions assessing depression and anxiety. Correlation matrices and bootstrapped CIs are reported in supplementary information (**Table S2**, **Figures S1** and **S2**).

### TAAR1 Missense Variants Are Enriched in Patients

We detected 21 SNPs/SNVs, of which 5 in up- or down-stream untranslated regions, and 16 in TAAR1 coding region. Among the latter, 13 were missense SNVs, with a MAF ranging from 0.002 to 0.006 in our sample, consistently with the MAF < 0.05 reported in public database: 11 occurred in patients (K15R, R23C, L32V, F95I, Y123S, Y131C, A168T, E169D, E219D, C263R, V303G), 1 in controls (G181V), and 1 in both groups (H278Q). None of the detected SNVs has been previously described in cohorts of patients with mental disorders. R23C has been recently reported in a cohort of patients with impaired glucose and weight control (Muhlhaus et al., 2017). Pooling all the missense SNVs, we found a significant enrichment of SNVs in the patient group (n = 11, 10.6% *vs.* n = 2, 1.5%, OR = 7.57, 95% CI 1.64–34.97, p < 0.01). Details about all the detected SNVs are shown in **Table 1**.

**Table 1:** TAAR1 single-nucleotide polymorphisms/variants in patients and controls and corresponding minor allele frequencies.

Chr	Position	SNP/SNV	Wild-type allele	Variant allele	mRNA (NM138327.2)	Protein (NP_612200.1)	WT/He/Ho in cases	MAF in cases	WT/He/Ho in controls	MAF in controls	MAF in different populations			
											Asia	Europe	Africa	Global
chr6	132646114	rs893620502	T	C	N/A	2-kb upstream	103/1/0	0.0048	130/0/0	0	–	–	–	–
chr6	132646091	–	C	A	N/A	2-kb upstream	104/0/0	0	129/1/0	0.0038	–	–	–	–
chr6	132645960	rs1199116910	T	C	60	K15R	103/1/0	0.0048	130/0/0	0	–	–	–	0.00001
chr6	132645937	rs8192618	G	A	90	R23C	103/1/0	0.0048	130/0/0	0	0	0	0.014	0.0016
chr6	132645910	–	G	C	117	L32V	103/1/0	0.0048	130/0/0	0	–	–	–	–
chr6	132645721	–	A	T	306	F95I	103/1/0	0.0048	130/0/0	0	–	–	–	–
chr6	132645636	rs371440762	T	G	391	Y123S	103/1/0	0.0048	130/0/0	0	0	0.0004	0	0.0002
chr6	132645612	rs41286174	T	C	415	Y131C	103/1/0	0.0048	130/0/0	0	–	–	–	–
chr6	132645503	rs753568048	C	T	525	A168T	103/1/0	0.0048	130/0/0	0	0	0	0	0.00001
chr6	132645497	–	T	A	530	E169D	103/1/0	0.0048	130/0/0	0	–	–	–	–
chr6	132645463	rs200695328	C	A	565	G181V	104/0/0	0	129/1/0	0.0038	0	0.0003	0	0.00017
chr6	132645348	–	T	G	679	E219D	102/2/0	0.0096	130/0/0	0	–	–	–	–
chr6	132645217	rs142169206	A	G	810	C263R	103/1/0	0.0048	130/0/0	0	0	0	0.0001	0.00001
chr6	132645209	rs8192619	G	A	818	C265-	98/6/0	0.0288	118/12/0	0.0462	0.093	0.052	0.06	0.064
chr6	132645170	–	G	C	857	H278Q	102/2/0	0.0096	129/1/0	0.0038	–	–	–	–
chr6	132645140	rs8192620	T	C	887	V288-	68/34/2	0.1827	100/26/4	0.1308	0.271	0.229	0.126	0.223
chr6	132645096	rs1045972396	A	C	931	V303G	103/1/0	0.0048	130/0/0	0	–	–	–	–
chr6	132645068	rs8192621	T	C	959	R312-	98/6/0	0.0288	121/9/0	0.0346	0.081	0.019	0.006	0.032
chr6	132644955	rs41286172	G	A	1072	3’utr	100/4/0	0.0192	128/2/0	0.0077	0.009	0.026	0.004	0.02
chr6	132644904	rs9375907	G	T	1123	3’utr	68/34/2	0.1827	100/26/4	0.1308	0.341	0.224	0.055	0.18
chr6	132644895	–	A	C	N/A	500 B downstream	104/0/0	0	129/1/0	0.0038	–	–	–	–

Chr, chromosome; He, heterozygous genotype; Ho, homozygous variant genotype; MAF, minor allele frequency; N/A, not applicable; WT, wild-type; –, no information available.

Six of the missense SNVs map in highly conserved positions (similarity ≥ 80% to identity) across several TAAR1 orthologous and other members of the human TAAR family (**Figure S3**). Of these, four (R23C, Y131C, C263R, V303G) were predicted to have a functional effect by *in silico* tools, and they all occurred in patients. We here selected a first subset of three SNVs—two new [Y131C (rs41286174) and C263R (rs142169206)] and the recently reported mutant R23C (rs8192618) (Muhlhaus et al., 2017)—to be tested on their functional effects *in vitro*. Each of these SNVs was observed in one heterozygous carrier, thereby corresponding to a MAF of 0.005, substantially higher than the MAF in the general population (**Table 1**). The psychopathological phenotype of the three carriers can be found in supplementary discussion. In brief, one of them suffered from schizoaffective disorder, and the other two from bipolar disorders.

### Detected TAAR1 Variants Are Located in Functional–Structural Key Regions

**Figure 2:**
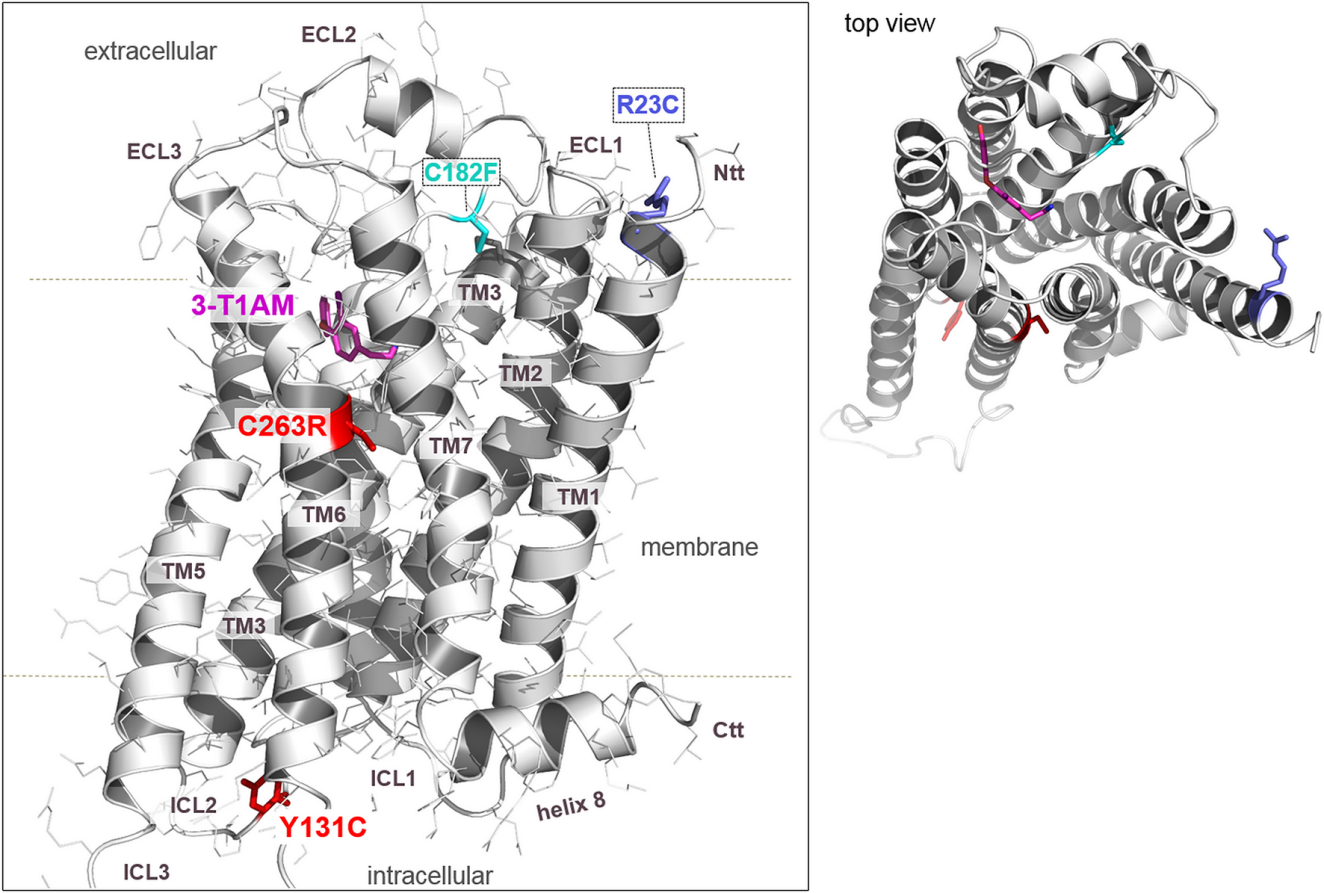
A homology model of human TAAR1 bound with T1AM reveals deeper insights into molecular pathogenic mechanisms of identified and tested naturally occurring substitutions. The hTAAR1 model (backbone representation) shows the spatial localization of the wild-type amino acids (colored sticks) at the positions of tested substitutions. As already known, Arg23 is located at the transition between TMH1 and the N-terminal tail (Ntt) at the extracellular side. The side chain of Arg23 can potentially interact with Asp21 or with partners in the extracellular loop 1 (ECL1) such as the negatively charged Glu86 (not shown). The amino acid Arg23 is conserved among TAAR1 orthologous (see **Figure S3**). In contrast, Tyr131 points to the cytosol between helix 5 and 6, which is known to be the G-protein or arrestin binding region. Cys263 is part of the TMH6 in the conserved CWxP motif of class A GPCRs. The site view on TAAR1, in combination with the top-view (insert), shows that none of the tested substitutions (R23C, Y131C, C263R) directly impact ligand binding (T1AM). The three variants analyzed in the present work (R23C, Y131C, C263R) are likely to affect protein stability and/or functional activity, including G-protein binding capacities. Of note, the previously described C182F mutation (John et al., 2017) interrupts the highly conserved disulfide bridge between the ECL2 and TMH3. Structure images were produced using the PyMOL Molecular Graphics System, Version 1.5, Schrödinger, LLC.

As shown in **Figure 2**, the here identified and tested TAAR1 variants are located spatially at different regions of the protein. First, Y131C is part of the second intracellular loop (ICL2) and the aromatic side chain points into the cytoplasmic crevice between the helices, which is the binding region for G-protein or arrestin molecules. Secondly, the C263R variant is in the transmembrane helix (TMH) 6, which is a) known to be essential for receptor activation and b) highly conserved (∼70%) at this position among class A GPCRs as part of the so called CWxP motif (Biebermann et al., 2012; Olivella et al., 2013). The cysteine is tightly packed in a hydrophobic environment between TMH6 and TMH7. In conclusion, the substitution cysteine to arginine likely causes tremendous modification of an essential hydrophobic patch in the TMH6–TMH7 interface reasoned by the positively charged, large, and bulky side-chain of arginine. As already described, R23C is located at the transition between TMH1 and the N-terminal tail at the extracellular side and could interact with important residues of the ECL1 (Muhlhaus et al., 2017).

### TAAR1 Variants R23C, Y131C, and C263R Decrease Cell Surface Expression

**Figure 3:**
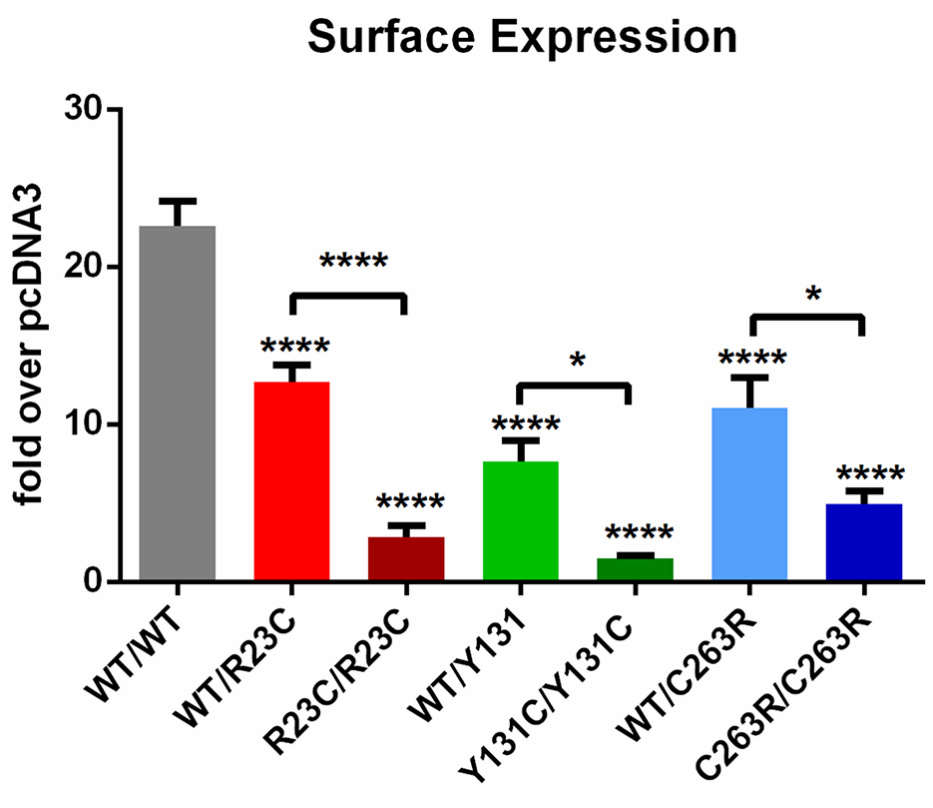
Cell surface expression of TAAR1 wild-type and TAAR1 variants. HEK293 cells were transiently transfected with (left to right) TAAR1-WT/WT, TAAR1-WT/R23C, TAAR1-R23C/R23C, TAAR1-WT/Y131C, TAAR1-Y131C/Y131C, TAAR1-WT/C263R, and TAAR1-C263R/C263R and N-terminally tagged with the HiBiT. GLP1R surface expression was measured as positive control (31.48 ± 2.67 RLU). Bioluminescence was detected upon application of the cell-impermeable LgBiT and luciferase substrate. The signal from each well was normalized over pcDNA3 (1.00 ± 0.01 RLU). Data are represented as mean ± SEM of fold over basal pcDNA3 and are based on four independent experiments performed in triplicates. *p ≤ 0.05 and ****p ≤ 0.0001 by one-way ANOVA and Dunnett’s *post hoc* test.

All three SNVs significantly altered TAAR1 cell surface expression [F(6,86) = 37.69, p < 0.0001], partially in the heterozygous state and more substantially in the homozygous state (**Figure 3**). In particular, cell surface expression of: i) TAAR1-WT/R23C was reduced to 56.08% of TAAR1-WT/WT (12.68 ± 1.12 *vs.* 22.61 ± 1.58, mean diff. = 9.93 ± 1.62, p < 0.0001); ii) TAAR1-WT/Y131C to 33.94% (7.67 ± 1.29, mean diff. = 14.93 ± 1.72, p < 0.0001); and iii) TAAR1-WT/C263R to 48.83% (11.04 ± 1.91, mean diff. = 11.57 ± 1.72, p < 0.0001). This effect was not due to a reduction in total TAAR1 expression. Indeed, none of the SNVs significantly altered total TAAR1 expression, as compared to TAAR1-WT (**Figure S4A**). The comparison of cell surface expression and total expression revealed that the fraction of TAAR1 on the cell surface was significantly reduced for all the three SNVs (**Figure S4B**), in detail: i) TAAR1-R23C was reduced to 42.07% of TAAR1-WT (60.75 ± 10.03% *vs.* 25.56 ± 5.99%, mean diff. = 35.18%, p ≤ 0.01), ii) TAAR1-Y131C to 30.44% (18.49 ± 3.77%, mean diff. = 42.26%, p ≤ 0.001), and iii) TAAR1-C263R to 54.86% (33.33 ± 3.74%, mean diff. = 27.42%, p ≤ 0.05).

### TAAR1 Variants R23C, Y131C, and C263R Dampen Gs Signaling

**Figure 4:**
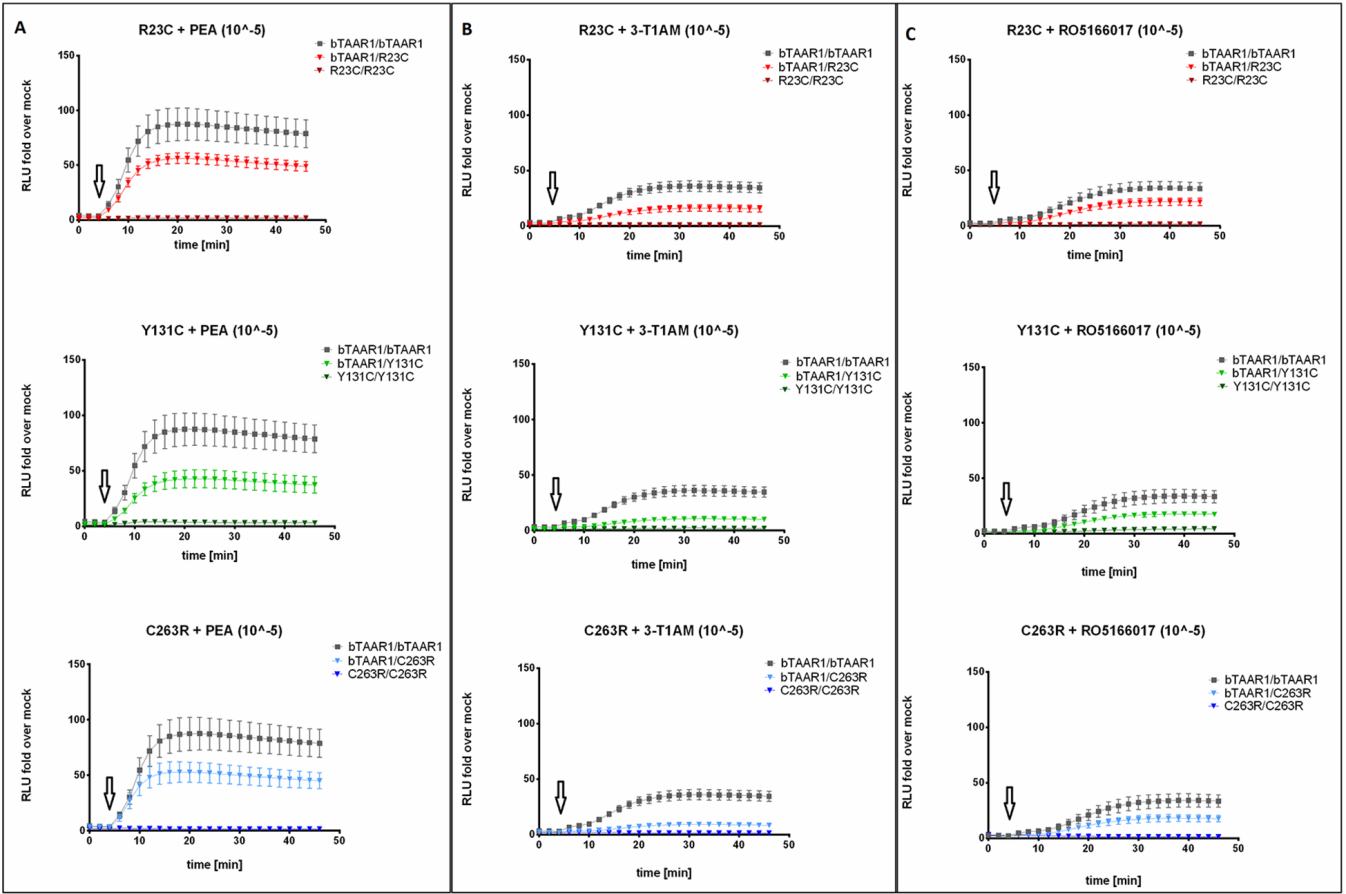
Gs signaling properties of TAAR1 wild-type and TAAR1 variants. HEK293 cells were transiently transfected with (top to bottom): TAAR1-WT/WT, TAAR1-WT/R23C, TAAR1-R23C/R23C, TAAR1-WT/Y131C, TAAR1-Y131C/Y131C, TAAR1-WT/C263R, TAAR1-C263R/C263R, and stimulated with **(A)** PEA 10 μM, **(B)** T1AM 10 μM, and **(C)** RO5166017 10 μM. Arrows indicate ligand stimulation. Real-time cAMP formation in live cells was measured as the increase in luminescence activity (relative light units, RLU). Data are represented as mean ± SEM of fold over basal of the empty vector (1,746 ± 238.4) and are based on five (PEA and T1AM) or four (RO5166017) independent experiments performed in triplicates.

HEK293 cells transfected with TAAR1-WT and variants were stimulated with 10 µm PEA, T1AM, and RO5166017. Formation of cAMP was monitored in living cells, and luciferase activity was measured as RLUs. The situation of a homozygous WT condition (TAAR1-WT/WT) was compared with the heterozygous condition (TAAR1-WT/variant) and the artificial homozygous variant situation (TAAR1-variant/variant). Following stimulation of TAAR1-WT/WT with PEA 10 μm, we observed a time-dependent increase in RLUs, which peaked after approximately 10-min stimulation, and accounted for a mean total AUC of 2,808 (95% CI 1,787–3,828) (**Figure 4A**). The maximal response of TAAR1-WT/WT to T1AM 10 μm was lower, with a mean total AUC of 1,024 (95% CI 746.4–1,302) (**Figure 4B**). In both cases, TAAR1-WT/WT appeared to promote sustained Gs signaling (**Figures 4A, B**).

TAAR1-R23C, TAAR1-Y131C, or TAAR1-C263R in homozygous condition showed a complete loss of function both in terms of basal activity (PBS stimulation, **Figure S5**) and in response to 10 μM PEA or T1AM (**Figures 4A, B**). In heterozygosity, all variants significantly reduced the mean total AUC upon stimulation with PEA 10 μM, without affecting the latency to the peak (TAAR1-WT/R23C, 63.57%, mean diff. = 1,023 ± 440.5, p < 0.05; TAAR1-WT/Y131C, 48.39%, mean diff. = 1,452 ± 463.8, p < 0.01; TAAR1-WT/C263R, 61.47%, mean diff. = 1,082 ± 471.8, p < 0.05) (**Figure 4A**). More robust AUC reductions followed stimulation with T1AM 10 μM (TAAR1-WT/R23C, 44.45%, mean diff. = 569 ± 134.7, p < 0.001; TAAR1-WT/Y131C, 29.59%, mean diff. = 721.2 ± 125.6, p < 0.0001; TAAR1-WT/C263R, 26.31%, mean diff. = 754.8 ± 122.3, p < 0.0001) again with no shifts in the latency to peak (**Figure 4B**). These results were mirrored by the assessment of cAMP accumulation with AlphaScreen technology, reported in **Figure S6**.

We tested whether RO5166017, a high-affinity and high-selectivity TAAR1 agonist, was able to restore Gs signaling of TAAR1 variants to the level of TAAR1-WT. The response to RO5166017 10 μM had the same effect as T1AM at TAAR1-WT/WT (AUC = 844, 95% CI 482.4–1,206 (**Figure 4C**). In addition, we observed no improvement of the AUC in cells co-transfected with TAAR1-WT and TAAR1-Y131C and co-stimulated with PEA 10 μM and RO5166017 10 μM, as compared to the PEA stimulation alone (**Figure S7**).

## Discussion

Mental disorders are characterized by highly polygenic architectures, involving common and rare variants, and many pleiotropic genes (Smoller et al., 2019). Recently developed heritability methods demonstrate a high degree of genetic overlap among mental disorders (Anttila et al., 2018), which corresponds to largely shared transcriptional dysregulations (Gandal et al., 2018). Given the profoundly interconnected nature of mental disorders, it is unlikely that the current diagnostic boundaries reflect distinct underlying pathogenic processes, at least at the genetic level (Anttila et al., 2018; Smoller et al., 2019). Therefore, for the present screening of TAAR1 variants, we recruited patients suffering from any major mental disorders, rather than focusing on a specific diagnostic category. In the human genome, the nine members of the TAAR family cluster on chromosome 6 at band q23.2. Genome-wide association studies (GWAS) have identified significantly enriched signals at chromosome 6 in schizophrenia and bipolar disorder (Burchett and Hicks, 2006; Schizophrenia Working Group of the Psychiatric Genomics, 2014). Also, SNPs in genes of the TAAR family (TAAR8, TAAR6) have emerged from GWAS investigating genetic variants associated with brain development (Szekely et al., 2018). As for TAAR1, a missense SNV (C182F) was detected in one affected mother and two affected children in a schizophrenia family, and Sanger sequencing screening of TAAR1 coding region revealed missense SNVs to be significantly enriched among 475 patients with sporadic schizophrenia, as compared to 410 healthy controls (John et al., 2017).

Consistently, here we confirmed a significant enrichment of missense SNVs in TAAR1 coding sequence. As a matter of fact, 11 out of 13 missense SNV occurred in patients, including all the variants predicted to have functional impact by means of *in silico* prediction tools. However, relying only on the latter is a rather improper procedure, because backgrounds of such predictions (related basic information) are incomplete. Therefore, we used these tools just as an initial screening to select a subset of detected amino acid side chain substitutions—R23C, Y131C, and C263R—to be experimentally tested for their effects. These three SNVs were found in one heterozygous carrier with schizoaffective disorder, and two heterozygous carriers with bipolar disorders. In addition, we inspected a 3D-molecular homology model to estimate putative molecular roles of WT amino acids and subsequent impact of variants.

All tested TAAR1 variants—namely, R23C, Y131C, and C263R—were characterized by decreased cell surface expression levels compared to TAAR1-WT. Since the overall expression of all three variants was not significantly reduced, a faster recycling process due to unstable protein can be excluded. Moreover, the comparison of total and cell surface expression revealed a noticeable reduction of the fraction of TAAR1 on the cell surface in all three cases. Although the subcellular localization of TAAR1 is still a matter of debate (Borowsky et al., 2001; Berry, 2004; Barak et al., 2008; Espinoza et al., 2011; Harmeier et al., 2015), decreased plasma membrane expression is expected to reduce the response to extracellular messengers.

By measuring cAMP formation in living cells, we demonstrated that the tested SNVs result in a partial loss of function after PEA (range: 48 to 64% of TAAR1-WT activity) and T1AM (range: 26 to 44% of TAAR1-WT activity) challenge in heterozygous state. Of note, TAAR1 basal activity is affected as well, when the SNVs are in homozygous conformation (**Figure S5**). The latter finding may have pathophysiological implications as well, since all vertebrate TAAR1 orthologs are known to be constitutively active (Coster et al., 2015).

These results are consistent with insights from our 3D TAAR1 model (**Figure 2**), which indicates that the tested TAAR1 variants are spatially located at essential hot spots for receptor functions. Y131 points into the intracellular crevice between the helices, in a key region for coupling of the receptor with G-protein after agonist binding (Krishna Kumar et al., 2019). The Y131C variant might modify the shape and biophysical properties of this crevice, by replacing the tyrosine bulky and aromatic ring with the cysteine aliphatic short side chain. Moreover, the tyrosine is characterized by a hydroxyl group which may establish intra- or intermolecular hydrogen bonds. The C263R variant—hydrophobic to hydrophilic and charged modification—likely interrupts the hydrophobic interface between TMH6 and TMH7, with a putative negative impact on TAAR1 signaling capacity, as both helices are obligatory involved in activation-related receptor modifications (Rose et al., 2014; Wootten et al., 2018). Such functional effect of a C to R substitutions at a corresponding position was previously reported for the thyrotropin receptor (TSHR) (Biebermann et al., 2012). Of note, this cysteine is part of the so called CWxP motif, which is a characteristic motif of class A GPCRs, strongly related to their activity regulation (Biebermann et al., 2012; Olivella et al., 2013). Further details on the molecular function of this conserved cysteine in GPCR activity state regulation are discussed in (Zhang et al., 2018b).

Recently, our laboratory identified and functionally characterized the R23C SNV in a cohort of patients with impaired glycemia and body weight regulation, with overlapping results. Besides the R23C SNV, another missense SNV, S49L, revealed to partially affect TAAR1 activity in that investigation (Muhlhaus et al., 2017). Strikingly, the two carriers presented mental health issues, respectively a borderline intellectual functioning (IQ = 71), and some not otherwise specified “psychiatric problems” (Muhlhaus et al., 2017). A bidirectional relationship exists between a broad range of mental disorders and diabetes (Ducat et al., 2014) and/or metabolic syndrome (Penninx and Lange, 2018). Partly related to unhealthy lifestyle and metabolic side effects of psychotropic drugs, this association may result from shared genetic vulnerability and pathophysiological mechanisms (Penninx and Lange, 2018), potentially including TAAR1. In fact, TAAR1 is highly expressed in brain areas involved in energy balance (Lindemann et al., 2008), in pancreatic islets, stomach, and gut, and has a role in insulin secretion, glucose homeostasis, and food intake (Regard et al., 2007; Raab et al., 2016). Interestingly, in rodents, TAAR1 agonists were shown to induce antipsychotic- and antidepressant-like activities, while controlling weight gain and fat accumulation, in contrast to most current standard—especially atypical—antipsychotics (Revel et al., 2013). Then, these compounds seem promising as a novel therapeutic option, to improve efficacy while reducing those undesirable side effects that negatively impact on patients’ quality of life, thereby representing an impediment to long-term compliance (Baptista et al., 2004).

In the present study, we were able to replicate our previous results using the GloSensor^™^ assay, which allows a real-time and live-cell kinetic and modulation analysis of signaling through cAMP. The temporal patterns of TAAR1 activation revealed sustained Gs signaling. This long-lasting action, common among GPCRs, may derive either from persistent signaling (Calebiro and Koszegi, 2019), activation of the downstream cascades after agonist binding, or from persistent agonist-independent activation of the receptor itself (Thomsen et al., 2016). Persistent activation may lead to prolonged influences on cell—particularly neuron—activity and synapse efficacy (Panaccione et al., 2013; Young et al., 2013).

The effect of the SNVs was more robust following T1AM, relative to PEA, stimulation. Interestingly, a recent study suggested that TAAR1 exerts different modulatory influences on other pathways depending on the identity of the stimulating agonist. In detail, T1AM, but not trace amines, was found to increase the activity of the tyrosine hydroxylase in the dorsal striatum, through a TAAR1-dependent phosphorylation of key regulatory sites (Ser19, Ser31, and Ser40). Therefore, disrupting the T1AM-TAAR1 interplay could specifically affect dopamine release, in brain areas involved in mood and psychotic disorders (Zhang et al., 2018a).

Of note, we observed qualitative differences in the spatiotemporal dynamics of Gs signaling at TAAR1-WT depending on the ligand (PEA *vs.* T1AM *vs.* RO5166017), suggesting that the phenomenon of biased signaling, already known for a wide array of GPCRs (Wisler et al., 2014), would extend to TAAR1. Binding of multiple ligands with diverse shape, size, and composition at the same site suggests that the receptor exists as an equilibrium between different conformational isomers, e.g., with different special relationships between its domains. In the binding process, the equilibrium is shifted toward the conformer with the most favorable geometry with respect to the incoming ligand (Ma et al., 2002). As for TAAR1, docking calculations suggested that T1AM, PEA, and RO5166017 share: 1) one H-bond between the amino groups and the D103 side chain; 2) π-π stacking interactions between the phenyl/ethylamino-phenyl rings and W264, F267, and F268. Additionally, RO5166017 oxazole ring also engages in a H-bond with T100, while the two oxygen atoms of T1AM interact with N268 and D287 through H-bonds (Cichero et al., 2013). Therefore, the three different ligands might stabilize different conformers, that in turn might lead to a different mode of activation, through either G-protein- or β-arrestin-dependent pathways, and eventually scaffold distinct downstream signaling molecules, such as kinases and phosphatases (Komatsu et al., 2019). A more complex picture may derive from the interrelation between TAAR1 and other GPCRs (Braunig et al., 2018; Kohrle and Biebermann, 2019). This evidence points to an elaborate signaling system, which warrants further investigations for its important physiological role and potential pharmaceutical applications.

Several limitations of our investigation should be acknowledged. First, we recruited a sample of relatively small size, for an investigation of SNVs of very low frequency. Nonetheless, we were able to find 13 missense SNVs, with a significant enrichment among patients, as compared to healthy controls. Also, the small sample size allowed us to deeply characterize our sample, in terms of main diagnosis, comorbidities, and psychopathological domains. Second, we performed the functional characterization of only three SNVs, which were selected according to their evolutionary conservation and the prediction of *in silico* tools. Given the shortcomings of such procedure, we are aware that, ideally, all the detected SNVs would deserve testing *in vitro* for their functional impact. Last, the response to endogenous and synthetic agonists was evaluated at a single dose, and no dose-response curve was performed. A deeper pharmacological characterization would also be necessary to unravel the implications of the different dynamics in TAAR1 response to the tested agonists.

In conclusion, our findings suggest that disruptions of TAAR1 activity may represent a vulnerability mechanism for the development of mental disorders. TAAR1 activity might be altered either because of missense mutations in the coding region, as in the present study, or due to decreased availability of its ligands. As T1AM is a putative thyroid hormone derivative, future studies should assess whether endocrine defects involving thyroid hormone can result in neuropsychiatric symptoms through a TAAR1-mediated mechanism. Furthermore, TAAR1 relevance for mental disorders provides a promising target for novel psychopharmacological interventions aimed at the treatment of these highly disabling disorders.

## Data Availability

The datasets generated for this study can be found in the dbSNP, PRJNA542354.

## Ethics Statement

The studies involving human participants were reviewed and approved by Comitato Bioetico dell’Università di Pisa, Protocol n° 55951, 12/09/2017. The patients/participants provided their written informed consent to participate in this study.

## Author Contributions

GR contributed to the conceptualization, formal analysis, investigation, visualization, and writing of the original draft. JB contributed to the formal analysis, investigation, methodology, supervision, visualization, review, and editing. CG contributed to the conceptualization, investigation, review, and editing. VC contributed to the conceptualization, formal analysis, investigation, review, and editing. IM, SM, LT, and SiP contributed to the investigation. GK contributed to the formal analysis, software, visualization, review, and editing. SaP contributed to the methodology, supervision, review, and editing. LD’O contributed to the resources. HB and RZ contributed to the conceptualization, funding acquisition, project administration, resources, supervision, review, and editing.

## Funding

This work is supported by the Deutsche Forschungsgemeinschaft, priority program 1629, BI893/5-2 to HB and PRA 2018 to RZ.

## Conflict of Interest Statement

The authors declare that the research was conducted in the absence of any commercial or financial relationships that could be construed as a potential conflict of interest.
