# Supplementary material for: Non-Functional Trace Amine-Associated Receptor 1 Variants in Patients With Mental Disorders
Source: Front Pharmacol. 2019 Sep 13;10:1027. doi: 10.3389/fphar.2019.01027 (PMC6753877; doi:10.3389/fphar.2019.01027)
Supplement: Supplementary file 1 [file DataSheet_1.docx]

**Supplementary material**

**Table S1. Current psychopathology scores**

| Scale | Median | 25th percentile | 75th percentile | Min | Max |
| --- | --- | --- | --- | --- | --- |
| PANSS total | 48.50 | 39.25 | 61.75 | 30 | 117 |
| *PANSS positive* | 9 | 7 | 11 | 7 | 38 |
| *PANSS negative* | 7.5 | 7 | 13 | 7 | 34 |
| *PANSS general* | 28 | 23.25 | 38 | 16 | 63 |
| YMRS total | 6 | 2 | 11.75 | 0 | 42 |
| *YMRS factor 1* | 1 | 0 | 3 | 0 | 10 |
| *YMRS factor 2* | 4 | 2 | 7 | 0 | 30 |
| *YMRS factor 3* | 0 | 0 | 1 | 0 | 11 |
| HDRS total | 7.5 | 4 | 12.75 | 0 | 26 |
| *HDRS depression* | 3.5 | 1 | 6 | 0 | 12 |
| *HDRS anxiety* | 2 | 1 | 5 | 0 | 12 |
| *HDRS insomnia* | 0 | 0 | 2 | 0 | 0 |
| *HDRS somatic* | 0 | 0 | 1 | 0 | 8 |

HDRS, Hamilton Rating Scale for Depression; PANSS, Positive and Negative Syndrome Scale; YMRS, Young Mania Rating Scale.

**Table S2. Correlation matrix in the psychopathology network**

|  | **PANSS positive** | **PANSS negative** | **PANSS general** | **YMRS**  **factor 1** | **YMRS**  **factor 2** | **YMRS**  **factor 3** | **HDRS**  **depression** | **HDRS anxiety** | **HDRS**  **insomnia** | **HDRS**  **somatic symptoms** |
| --- | --- | --- | --- | --- | --- | --- | --- | --- | --- | --- |
| **PANSS positive** | 1 |  |  |  |  |  |  |  |  |  |
| **PANSS negative** | -0.0433 | 1 |  |  |  |  |  |  |  |  |
| **PANSS general** | 0.2982 | 0.5348 | 1 |  |  |  |  |  |  |  |
| **YMRS**  **factor 1** | 0.6594 | -0.2970 | -0.0554 | 1 |  |  |  |  |  |  |
| **YMRS**  **factor 2** | 0.7381 | -0.1055 | 0.2839 | 0.7429 | 1 |  |  |  |  |  |
| **YMRS**  **factor 3** | 0.5282 | 0.1507 | 0.2953 | 0.2806 | 0.4243 | 1 |  |  |  |  |
| **HDRS**  **depression** | -0.0985 | 0.6863 | 0.6478 | -0.2646 | -0.1249 | 0.0519 | 1 |  |  |  |
| **HDRS anxiety** | 0.3428 | 0.1103 | 0.7170 | 0.1947 | 0.3879 | 0.1602 | 0.3163 | 1 |  |  |
| **HDRS**  **insomnia** | 0.4100 | 0.0848 | 0.3751 | 0.3265 | 0.3829 | 0.0748 | 0.1010 | 0.4518 | 1 |  |
| **HDRS**  **somatic symptoms** | -0.1077 | 0.1282 | 0.3422 | -0.0809 | -0.0838 | -0.0334 | 0.2843 | 0.4213 | 0.2625 | 1 |

HDRS, Hamilton Rating Scale for Depression; PANSS, Positive and Negative Syndrome Scale; YMRS, Young Mania Rating Scale.

**Figure S1. Bootstrapped confidence intervals of estimated edge-weights for the estimated network of PANSS, YMRS and HDRS scores.** The red line indicates the sample values, the black line indicates the bootstrap sample values, and the gray area the bootstrapped CIs. Each horizontal line represents one edge of the network, ordered from the edge with the highest edge-weight to the edge with the lowest edge-weight. The y-axis labels have been removed to avoid cluttering. Sizable bootstrapped CIs around the estimated edge-weights indicate that there is no significant difference between many edge-weights. The large bootstrapped CIs suggest care in the interpretation of the order of most edges in the network. The 4 strongest edges are: PANSS general - HDRS anxiety; YMRS factor 1 - YMRS factor 2; PANSS positive - YMRS factor 2; PANSS general – HDRS depression.


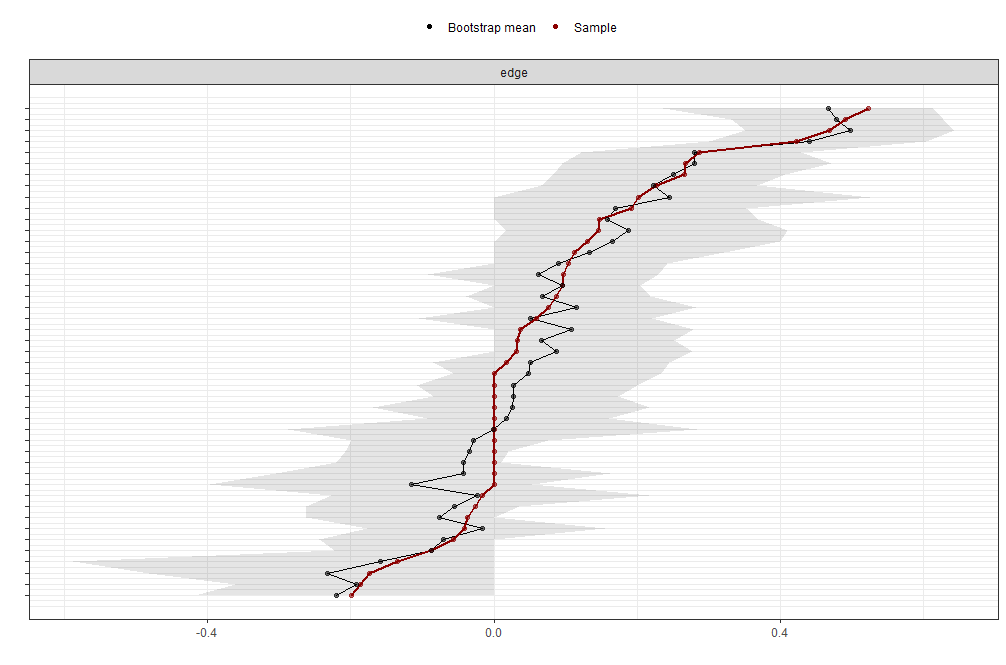


**Figure S2. Average correlations between centrality indices of networks sampled with persons dropped and the original sample.** Lines indicate the means and areas indicate the range from the 25th to the 75th percentile. Central stability coefficient (maximum drop proportions to retain correlation of 0.7 in at least 95% of the sample) for the psychopathology network was 0.125 for betweenness, 0.202 for closeness, and 0.202 for strength.


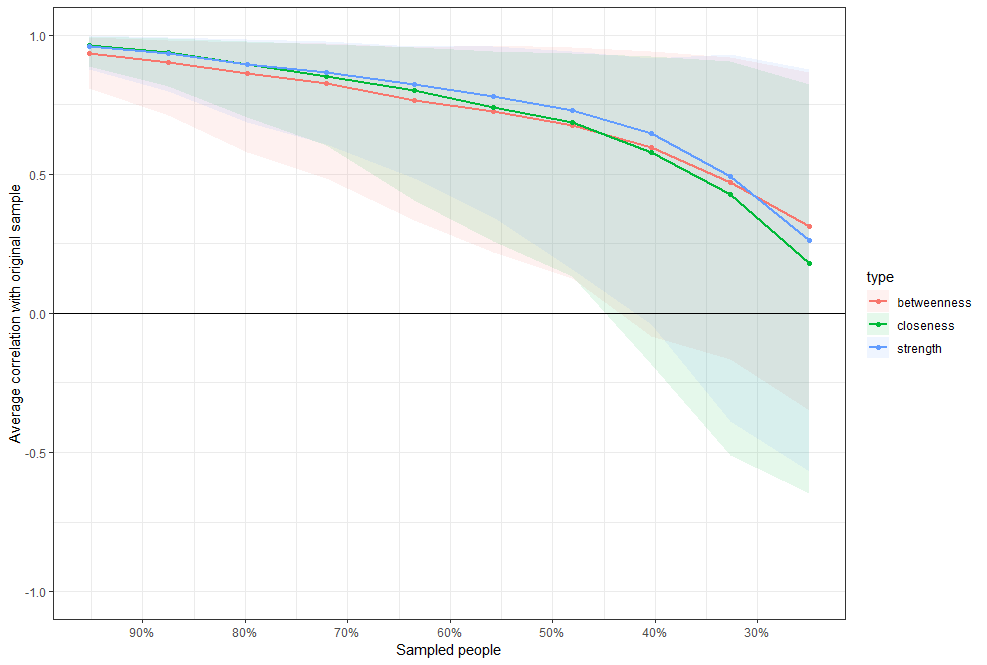


**Figure S3. Sequence alignment of human TAAR1 with orthologue genes across several species and paralogue genes in the TAAR family.** The alignment was performed manually and visualized with the software BioEdit. Amino acid residues showing higher-than-80% similarity to identity, indicating evolutionary conservation (Blosum62 matrix), were shaded with different colors, according to the biophysical properties of the amino acid side chains, as follows: positively charged (R, K), blue; aromatic and hydrophobic (A, F, Y, W), cyan/light green; hydrophobic (V, I, L, M), dark green; negatively charged (D, E), red; polar uncharged (S, T, N, Q), gray; H, magenta; C, dark red; G, orange; P, brown. The dots indicate residue identity throughout the aligned sequences. Missense variants in residues with <80% similarity are reported in black boxes. Missense variants in highly conserved residues are reported in blue boxes, if found among patients, or in red boxes, if found among healthy controls.

**
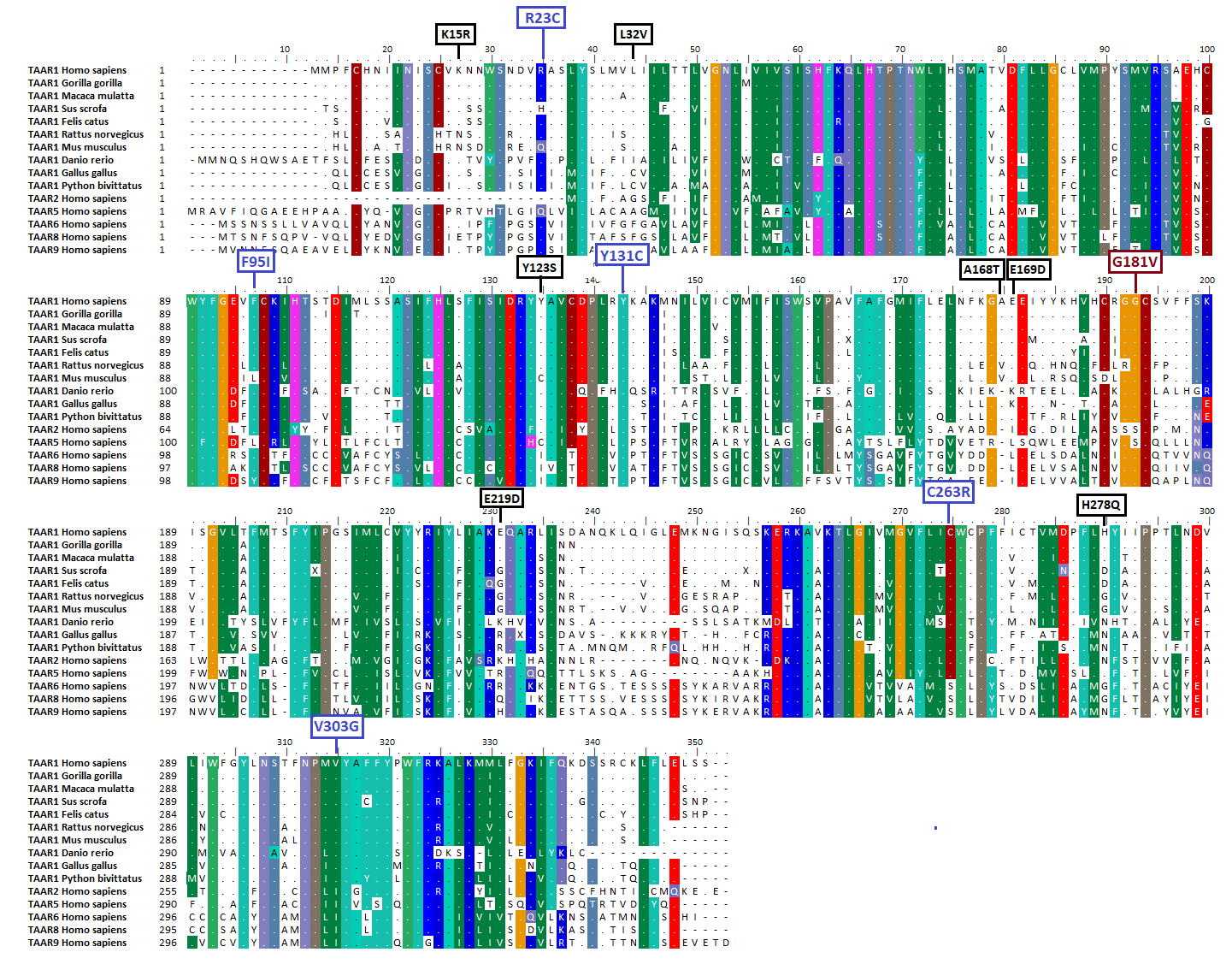
**

**Supplementary Discussion**

The carrier of the R23C variant was a 28-year-old man, affected by SZA, bipolar type, and co-morbid OCD. He reported a family history of SCZ spectrum disorders. First appearance of psychotic symptoms occurred at 18 years of age. At the recruitment, he presented with a mixed affective state with moderate depressive symptoms, very severe psychotic symptoms, particularly delusions, high levels of anxiety, worry, inner tension and restlessness, and obsessive-compulsive symptoms. There were no suicide attempts in his psychiatric history.

The carrier of the Y131C variant was a 54-year-old man, with family history of major depressive and anxiety disorders and prenatal abnormalities, namely umbilical cord compression-derived hypoxia. At the age of 52, he was admitted to hospital with excitement, irritability, agitation, paranoid thoughts, bizarre and disorganized behavior and speech, and given a diagnosis of BD-I. He had also gradually developed blunted affect, avolition, social withdrawal, cognitive deficits over the last months. Noteworthy, he later received a diagnosis of frontotemporal dementia, behavioral variant.

The carrier of the C263R variant was a 60-year-old woman, suffering from BD-II since the age of 35. She had family history for mental disorders, unspecified. At recruitment, she presented with a severe depressive episode characterized by psychomotor retardation, loss of will, energy and pleasure, feeling of worthlessness and guilt, worry, poor concentration, social withdrawal, insomnia, thoughts of death and low to mild anxiety. She reported no previous suicide attempts.

Here follows a table summarizing the scores obtained by the 3 carriers in the psychopathology scales.

| Scale | R23C | Y131C | C263R |
| --- | --- | --- | --- |
| PANSS total | 73 | 117 | 56 |
| *PANSS positive* | 15 | 29 | 7 |
| *PANSS negative* | 11 | 34 | 11 |
| *PANSS general* | 47 | 54 | 38 |
| YMRS total | 14 | 28 | 0 |
| *YMRS factor 1* | 1 | 5 | 0 |
| *YMRS factor 2* | 11 | 14 | 0 |
| *YMRS factor 3* | 2 | 7 | 0 |
| HDRS total | 16 | 17 | 23 |
| *HDRS depression* | 7 | 7 | 11 |
| *HDRS anxiety* | 9 | 6 | 5 |
| *HDRS insomnia* | 0 | 4 | 5 |
| *HDRS somatic* | 0 | 0 | 2 |

HDRS, Hamilton Rating Scale for Depression; PANSS, Positive and Negative Syndrome Scale; YMRS, Young Mania Rating Scale.

**Figure S4. (A)Total protein expression of TAAR1 wild-type and TAAR1 variants.** HEK293 cells were transiently transfected with (left to right) TAAR1-WT, TAAR1-R23C, TAAR1-Y131C, TAAR1-C263R or GLP1R as positive control, N-terminally tagged with the HiBiT. Bioluminescence was measured after addition of the LgBiT protein and Nano-Glo® substrate in a lytic buffer to determine the total amount of HiBiT-tagged protein in the cell. The signal from each well was normalized over pcDNA3 (1.00 ± 0.01 RLU). Data are represented as mean ± SEM of fold over basal pcDNA3 and are based on four independent experiments performed in triplicates. **(B) Fraction of TAAR1 wild-type and TAAR1 variants on the cell surface in comparison to total expression.** To compare total and cell surface expression, the measurement of cell surface expression was performed simultaneously with the total expression in (A). Bioluminescence was measured after the addition of the LgBiT protein and Nano-Glo® substrate in a non-lytic buffer to solely determine the expression on the cell surface. A control protein that is 100% extracellular was used in both assays to compare signals between the two buffer conditions and determine the fraction of protein on the cell surface (%). Data are represented as mean ± SEM of % of totally expressed receptor and are based on four independent experiments performed in triplicates. ∗p ≤ 0.05 and ∗∗∗p ≤ 0.001 by one-way ANOVA and Dunnet’s post-hoc test.


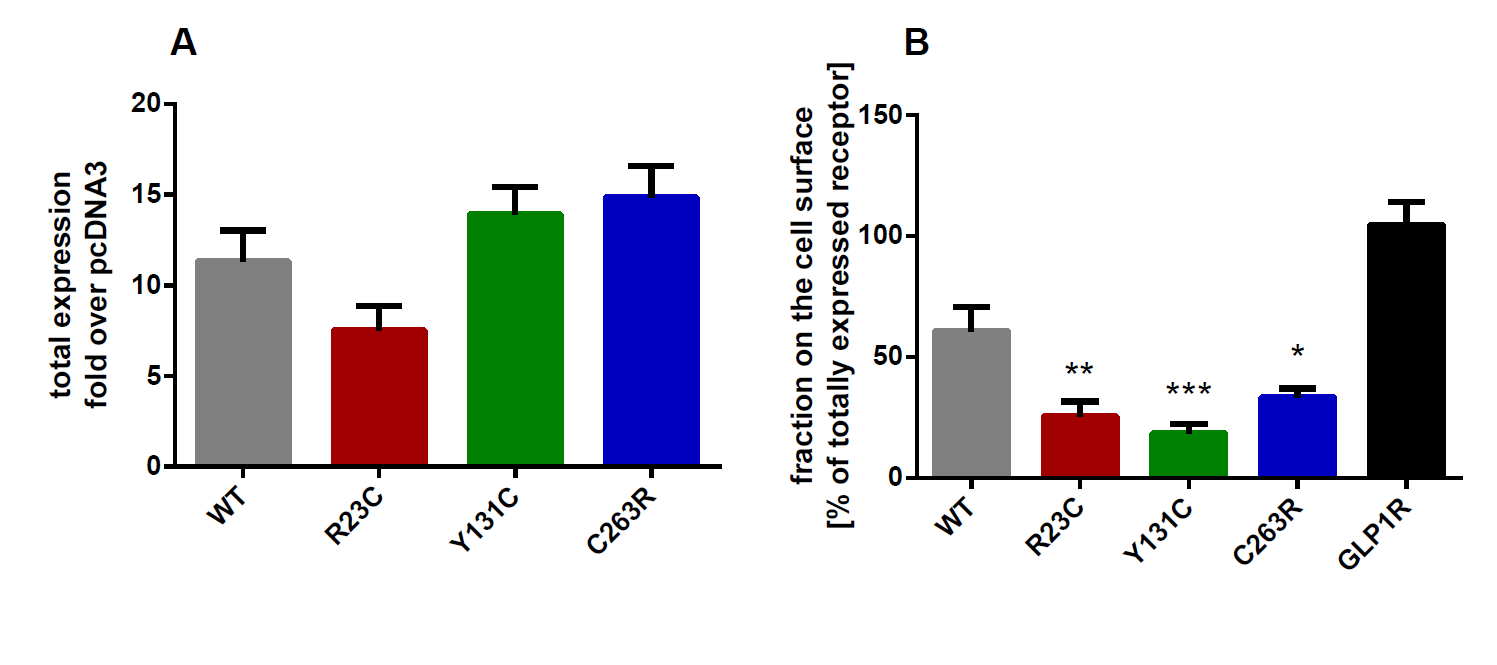


**Figure S5. Assessment of TAAR1 Gs signaling with the GloSensor^TM^ cAMP assay. (A) Negative controls.** HEK293 cells were transiently transfected with: TAAR1-WT/WT, TAAR1-WT/R23C, TAAR1-R23C/R23C, TAAR1-WT/Y131C, TAAR1-Y131C/Y131C, TAAR1-WT/C263R, TAAR1-C263R/C263R, and stimulated with PBS. (B) **Positive controls.** HEK293 cells, known to endogenously express β-adrenergic receptors, were transiently transfected with the pcDps empty vector and stimulated with isoproterenol. Real-time cAMP levels in live cells was measured as the increase in luminescence activity (relative light units, RLU). Data are represented as mean ± SEM of fold over basal of the empty vector.





**B**

**A**

**Figure S6. Assessment of Gs signaling properties of TAAR1 wild-type and variants with the AlphaScreen technology.** HEK293 cells were transiently transfected with TAAR1-WT/WT, -WT/R23C, -WT/Y131C, and -WT/C263R, and stimulated with T1AM (10^-7^ M – 10^-5^ M). cAMP accumulation was measured with the AlphaScreen technology. Cells transfected with empty vector (pcDps) served as a negative control. Data are represented as mean ± SEM of fold over basal of the respective receptor variant and are based on 3 independent experiments performed in triplicates.





**Figure S7. Gs signaling properties of TAAR1 wild-type and the TAAR1 variant Y131C in heterozygous conformation upon co-stimulation with PEA and RO5166017.** (A) HEK293 cells were transiently transfected with TAAR1-WT/WT or TAAR1-WT/Y131C, and co-stimulated with PEA 10 μM, or the combination of PEA 10 μM and RO5166017 10 μM. Real-time cAMP levels in live cells was measured as the increase in luminescence activity (relative light units, RLU). Data are represented as mean ± SEM of fold over basal of the empty vector (not shown) and are based on 3 independent experiments performed in triplicates. (B) We observed no improvement of the AUC in cells co-transfected with TAAR1-WT and TAAR1-Y131C and co-stimulated with PEA 10 μM and RO5166017 10 μM, as compared to the PEA stimulation alone. Indeed, for the PEA- RO5166017 co-stimulation, an AUC of 308.7 was calculated, that corresponded to the 50.76% of response of cells transfected with TAAR1-WT/WT to PEA 10 μM (mean diff. 299.5 ± 118, p < 0.05).





**B**

**A**
